# Volumetric motion quantification by 3D velocity encoded MRI

**DOI:** 10.1186/1532-429X-14-S1-P247

**Published:** 2012-02-01

**Authors:** Anja Lutz, Jan Paul, Axel Bornstedt, Gerd Ulrich Nienhaus, Patrick Etyngier, Peter Bernhardt, Wolfgang Rottbauer, Volker Rasche

**Affiliations:** 1 University Hospital of Ulm, Ulm, Germany; 2 Karlsruhe Institute of Technology, Karlsruhe, Germany; 3 Medisys Research Lab, Philips Healthcare, Suresnes, France

## Summary

It is objective to show the feasibility of volumetric velocity-encoded MRI (3D-TPM) to derive velocity based motion quantification parameters.

## Background

About 30% of patients treated with cardiac resynchronization therapy (CRT) do not benefit from the procedure. The introduction of quantitative parameters for selection of patients appears mandatory. However, parameters based on 2D imaging techniques may be less reproducible caused by rapid changes of the motion pattern along the cardiac axis.

## Methods

12 volunteers (26±7 years) and 2 patients (46, DCM/29, LBBB) were investigated at a 3T whole body MR scanner (Achieva, Philips) with a 32 channel cardiac coil. A 3D black blood velocity encoded navigated segmented gradient echo sequence (3D-TPM) was applied for whole heart coverage. The acquisition parameters were: FOV=380^2^mm^2^, isotropic resolution of 3x3x3mm^3^, FOV in through-plane direction =63mm in healthy volunteers (this FOV was increased in patients due to their enlarged heart corresponding to the distance between basis and apex), acquisition matrix =128x124, TR/TE=7.1ms/4.9ms, flip angle =15°, SENSE =4, nominal scan duration =15:30 minutes, 3 k-lines per segment, phase interval =37.3ms and 25 cardiac phases for 60bpm. The acquisition of the whole heart data was split into 3 chunks to ensure sufficient black blood contrast.

From the 3D-TPM data, the following parameters were evaluated: longitudinal and radial standard deviation of time to peak systolic and diastolic velocities SD(TTP_l,sys_), SD(TTP_l,dias_), SD(TTP_r,sys_), SD(TTP_r,dias_)[1], the mean radial, circumferential and longitudinal asynchrony correlation coefficient over all cardiac segments (ACC)[2], the longitudinal and radial velocity range Δv_l_ = v_l,max_-v_l,min_, Δv_r_ = v_r,max_-v_r,min_ and the new parameter temporal uniformity of velocity (TUV) in radial, longitudinal and circumferential direction, which was defined analogously to the temporal uniformity of strain defined in [3,4].

## Results

**Figure 1:**
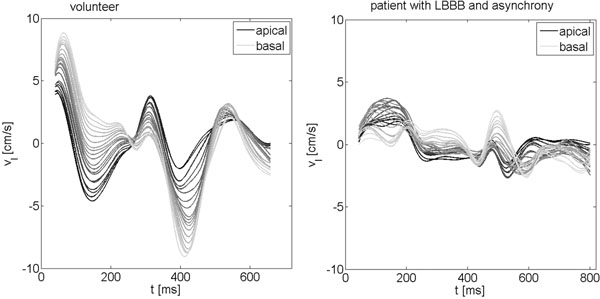
Velocity over time curves resulting from 3D TPM data exemplarily for one volunteer and one patient with LBBB and asynchrony.

Figure 1 shows the 3D velocity over time curves exemplarily for one volunteer and patient 2 with proven asynchrony. A clearly modified motion pattern with reduced peak velocities can be appreciated as well as the large variation of the motion curve along the heart axes in the volunteer. Table 1 contains the motion quantification parameter evaluation based on 3D-TPM data as mean ± standard deviation for the volunteer group and the patients. The mean ACC over all segments is decreased and the velocity ranges and TUV are decreased in the patients compared to healthy volunteers. SD(TTP_sys,r_) is increased in case of asynchrony.

**Table 1:** Velocity based motion parameters evaluated for all volunteers (mean ± standard deviation) and for investigated patients.

parameter	volunteers		patient 1	patient 2
	mean	σ	[DCM]	[LBBB, asynchrony]

SD(TTP)_l,sys_ [ms]	37.08	22.49	24.02	56.73

SD(TTP)_l,dias_ [ms]	19.52	3.78	6.04	54.10

SD(TTP)_r,sys_ [ms]	42.41	8.08	48.69	80.89

SD(TTP)_r,dias_ [ms]	37.90	7.44	26.00	51.40

mean ACC_l_	0.90	0.02	0.77	0.58

mean ACC_r_	0.71	0.06	0.55	0.40

mean ACC_c_	0.72	0.04	0.52	0.34

mean Δv_l_ [cm/s]	13.40	2.30	7.07	4.66

mean Δv_r_ [cm/s]	7.20	0.78	3.42	4.45

TUV_l_	0.86	0.01	0.74	0.74

TUV_r_	0.78	0.02	0.71	0.66

TUV_c_	0.77	0.03	0.69	0.62

## Conclusions

It is feasible to determine motion quantification parameters based on 3D velocity data. Huge differences could be observed between asynchronous patients and volunteers. A study including more patients is necessary to decide whether 3D -TPM data may in future distinguish between responders and non-responders to CRT.

## Funding

AL and VR have a research agreement with Philips Healthcare. PE is employed by Philips Healthcare.
